# Cochlear implantation outcomes in adults: A scoping review

**DOI:** 10.1371/journal.pone.0232421

**Published:** 2020-05-05

**Authors:** Isabelle Boisvert, Mariana Reis, Agnes Au, Robert Cowan, Richard C. Dowell

**Affiliations:** 1 The HEARing Cooperative Research Centre, Melbourne, Australia; 2 Faculty of Human Sciences, Audiology, H:EAR, Macquarie University, Sydney, Australia; 3 Faculty of Medicine and Health, The University of Sydney, Sydney, Australia; 4 Audiology and Speech Pathology, The University of Melbourne, Melbourne, Australia; 5 Cochlear Implant Clinic, The Royal Victorian Eye and Ear Hospital, Melbourne, Australia; Shinshu University School of Medicine, JAPAN

## Abstract

Cochlear implantation has become known as “the treatment of choice” for adults with severe to profound hearing loss. Wide variability exists, however, in the way hearing loss severity is measured and in the candidacy criteria used to recommend cochlear implantation.

**Objectives:**

This study aimed to provide a descriptive analysis of recent evidence available in the literature in relation to the efficacy of unilateral cochlear implantation in adults, the general findings of these studies, and the populations to which these findings apply. It also aimed to appraise the individual success rate and the magnitude of benefit following implantation.

**Design:**

A scoping review was conducted to identify English-language, peer-reviewed journal articles published between 2000 and 2018 assessing the outcomes of cochlear implantation in adults who received their first cochlear implant from 2000 onwards. To be included, studies had to report speech perception or self-reported measures of listening or quality of life at least three months after implantation. Systematic searches were conducted in Medline, Embase, Web of Science and Google Scholar. A two-stage screening approach was used, with seven reviewers independently screening titles and abstracts against inclusion criteria and three from this group further reviewing full-texts. A data charting form was developed and trialled, with 10% of the study data extracted in duplicate to compare results and further refine the form. Data relevant for efficacy analyses were extracted from studies with sample sizes of at least 10 participants.

**Results:**

A total of 4182 abstracts were screened against inclusion criteria, and of these, 603 full-texts were further screened. After exclusion of non-eligible articles, 201 articles were included in the first part of this scoping review. The majority of these articles were case series or comparative studies without a concurrent group, and had small sample sizes. Data synthesis conducted with the 102 articles with more than 10 participants highlighted that the average word perception ability improved from 8.2% to 53.9% after implantation. Self-reported benefit improved by 21.5 percentage points. At the individual level, 82.0% of adults with postlingual hearing loss and 53.4% of adults with prelingual hearing loss improved their speech perception ability by 15 percentage points or more. A small proportion had poorer ability after implantation or had stopped using the cochlear implant.

**Conclusions:**

Despite broad inconsistencies in measurement, research design, and reporting across articles, it is evident that cochlear implantation is beneficial to the majority of adults of any age who have limited aided speech perception abilities. While many adults with severe-to-profound hearing loss may also have poor speech perception abilities with hearing aids, the validity of using hearing loss severity as a criterion for cochlear implantation has not been demonstrated. Clinical and research recommendations derived from this review are provided.

## Introduction

The use of electrical stimulation of the auditory nerve to restore hearing dates back more than 60 years with the first medical report of auditory percepts in a deaf subject in 1957 [1]. Since that time, the rapid advances in materials science, electronics and digital technology have led to highly sophisticated electrode arrays, implanted electronics and sound processors that can deliver patterned auditory information at rapid rates to surviving auditory neurons within the cochlea. Research in psychophysical and speech sciences has guided the way this auditory information is delivered to the auditory system for optimizing the perception of speech sounds [2]. There remain, however, limitations to the quality of hearing that can be generated with these devices. Evidence has suggested that many factors related to device parameters, surgical placement of the implant, etiology, progression of hearing loss, and other patient-specific factors all play a role in the degree of benefit obtained [3–5]. This results in significant variability in outcomes across the population of cochlear implant recipients. For the individual candidate, the variability of outcomes with cochlear implants limits the degree of prognostic certainty that clinicians can provide when making recommendations on the potential for benefit [6]. The greater the degree of preoperative residual hearing, the greater the perceived potential for loss in the unlikely event of an unsuccessful intervention.

A further complicating factor is the contrast between assessing hearing using sound detection thresholds (the traditional hearing test), and the functional use of hearing for communication, generally demonstrated by measuring speech perception with various materials and under various conditions. Hearing threshold tests are useful when prescribing conventional hearing aids, but for those using cochlear implants, they provide little information about functional hearing for communication. Specifically, while audibility after implantation is restored within a relatively narrow range across the population, variability in speech perception outcomes is much broader and does not appear to relate directly to hearing thresholds. To a large extent, it is improvement in speech perception that is the primary goal of cochlear implantation, as this will in general facilitate improved communication and concomitant quality of life. Accordingly, cochlear implantation outcomes are generally assessed using a number of speech perception tests along with measures of quality of life, which can be broadly defined as the subjective experience of both positive and negative aspects of life across different domains.

The relative success of cochlear implantation in restoring functional communication has seen an expansion of their application from the relatively small population of totally deaf individuals with little or no residual hearing, to the much larger population of people with cochlear hearing loss who have difficulties understanding speech with acoustic hearing aids in one or both ears [7, 8]. Along with advances in device technology and surgical techniques that aim to preserve residual hearing, this means that a wider population of patients can potentially benefit from cochlear implantation. What remains difficult to determine is a clear set of implantation candidacy criteria—that is, the point at which a cochlear implant would be expected to provide better functional hearing over acoustic hearing devices.

Within the literature, cochlear implantation is frequently referred to as the “standard of care” [9, 10], “treatment of choice” [11, 12], or the “gold standard” for management of patients with severe-to-profound sensorineural hearing loss [13] perhaps based on the common belief that patients with severe-to-profound hearing loss cannot derive benefit from traditional hearing aids [c.f. 14]. Such broad statements become problematic, however, when definitions and measures of hearing loss severity are inconsistent across studies. Even more problematic is the wide variability in the speech recognition ability of patients with severe-to-profound hearing loss when using hearing aids [15], contradicting the belief that they cannot benefit from a less invasive technology.

Cochlear implant candidacy is commonly assessed using aided speech perception scores as a representation of functional hearing difficulties, together with measures of hearing thresholds to confirm the diagnosis of hearing loss. However, a recent survey of cochlear implant candidacy criteria conducted across 20 countries revealed that guidance for implantation varied greatly and was largely related to the availability of funding, with publicly funded regions often applying more conservative audiometric criteria with little flexibility [16, 17]. As a result, access to cochlear implantation may be delayed for someone with poor speech perception scores but better hearing thresholds—even if functional hearing for communication could reasonably be improved with implantation. Raine and Vickers [16] also reported that a variety of speech perception tests were currently being used around the world to assess cochlear implant candidacy, ranging from monosyllabic and disyllabic words and phonemes to various measures of open-set sentence recognition. To further complicate matters, labelling indications on benefits from each device manufacturer also differ depending on regulatory requirements [e.g., Food and Drug Administration (FDA) approval; 7]. Sentence-based criteria are commonly used by manufacturers and in clinics in many countries [17], but their suitability for determining cochlear implantation candidacy has been debated given that they are prone to ceiling effects in quiet listening conditions, and tend to overestimate preoperative listening ability as a result. Gifford et al. [18] found that in a cohort of 156 cochlear implant recipients, 71% were able to score >85% correct on a sentence recognition in quiet test. In addition, subjects who scored 100% correct on the sentence test showed a wide range of performance from 20–94% correct on monosyllabic word recognition—a speech perception measure that does not suffer from ceiling effects in the population of interest. This raises questions about the sensitivity of sentence recognition scores in quiet as a criterion for assessing pre-implant listening ability, as well as its suitability for tracking post-implant auditory performance over time [18]. In response to these concerns, the use of self-reported hearing ability and quality of life measures has become more common during candidacy assessment and following implantation.

Given the relatively low penetration rates for cochlear implantation that have been reported in recent years [7, 19], it is likely that many adults with hearing impairment who may benefit from a cochlear implant have limited access to this technology due to restrictive or ambiguous candidacy criteria being used in many clinics [20]. A systematic approach to integrating current evidence with a focus on clinical applicability appears to be lacking, despite an abundance of published studies on the efficacy of unilateral cochlear implantation [c.f. 21]. This knowledge would support the further development of consistent and clinically aligned candidacy criteria.

This scoping review aimed to appraise and integrate recent evidence of the outcomes of cochlear implantation in adults. In particular, it aimed to provide an overview of:

1. the study design characteristics of recent sources of evidence;
2. the characteristics of the participants included in these studies (thus describing the population to which these findings apply); and
3. the efficacy and effectiveness of cochlear implantation at the individual and group level.

It is hoped that this knowledge will inform the design and reporting of future studies.

## Methods

This scoping review complies with the Preferred Reporting Items for Systematic Reviews and Meta-Analysis extension for Scoping Reviews [PRISMA-ScR; 22, see S1 Data], and the Australian code for responsible conduct of research.

### Protocol and registration

The protocol for this review was prospectively registered with PROSPERO International Prospective Register of Systematic Reviews (CRD42018089401) in July 2018 and is available online from: http://www.crd.york.ac.uk/PROSPERO/display_record.php?ID=CRD42018089401. This scoping review is part of a larger project examining predictive factors of cochlear implantation outcomes.

### Eligibility criteria

#### Types of studies

Eligible studies were English-language, peer-reviewed journal manuscripts published between 2000 and 2018 that assessed cochlear implantation outcomes in adults.

#### Types of participants

Eligible studies included those assessing adults (≥18 years of age) with hearing loss in either ear who received their first cochlear implant from 2000 onwards. Studies were excluded from the review if adult data was not reported separately from children data. Where only a portion of participants met the eligibility criteria and their results were reported individually, data from those eligible participants were included in the review.

#### Types of intervention

Eligible studies reported on outcomes of cochlear implant systems with full-length electrode arrays implanted from 2000 onwards to ensure that outcomes from comparable technologies were reported in the review. Studies reporting on date of implantation prior to 2000 were included only if data from 2000 onwards was reported separately. Other information was used to estimate the implantation date when it was not reported, including the release date of device models and the development date of outcome measures reported in the articles. When none of these were available, studies were excluded.

#### Types of outcome measure and timing

Outcome measures included behavioural speech perception tests performed in quiet or in noise with the first side implantation surgery, as well as formal self-report assessments of listening or quality of life (health-related or hearing specific). To be included, outcomes had to be assessed at least three months postoperatively. Speech perception outcomes for bilaterally implanted individuals were not included if separate ear outcomes were not reported.

### Information sources and search strategy

Searches were conducted in Medline, Embase, Web of Science, and Google Scholar; for the latter, only the first 200 search results were retrieved. The selection of databases was based on recommendations from Bramer et al. [23], which suggests that these databases represent the minimum requirement for adequate and efficient searches in systematic reviews. The most recent search was executed on 23 August 2018. The search strategy terms are provided in S2 Data. The search results for each database were exported into EndNote and then Covidence [24] for assessment of eligibility criteria.

### Selection of sources of evidence

Studies were screened in two stages. First, two of seven reviewers in the team (AA, AW, IB, JL, MA, MR, NS) independently screened study titles and abstracts against the inclusion criteria to determine their eligibility for inclusion in the review. Full texts of potentially relevant articles were then independently assessed for eligibility by two of three reviewers (AA, IB, MR). Where any conflicts arose regarding eligibility of studies, the three reviewers reached a final decision through discussion and consensus. The exclusion criteria for type of intervention was revised partway through the selection process to include studies that did not explicitly report on date of implantation if this information could be estimated based on device models or date of development of outcome measures. The risk of revising the protocol at the selection stage was minimised as this was modified after only one reviewer had commenced full text screening (see PROSPERO registration for differences between the protocols).

### Data charting process

A data-charting form was jointly developed by three reviewers (AA, IB, MR) in Microsoft Excel to determine variables to be extracted from studies. Following recommendations from Levac et al. [25], data from 10% of the studies were charted in duplicate and the results compared and discussed to refine the data-charting form and ensure that all relevant data was captured consistently amongst reviewers (AA, IB, MR). The remaining eligible studies were charted independently by each of the three reviewers. Data was extracted as reported in articles and study authors were not contacted when study information was unclear or not reported.

### Data items

Data charting categories included publication characteristics (e.g. year and journal of publication, country in which data was collected) and study characteristics (e.g. study design, number of participants with eligible data, onset of hearing loss, specific population characteristics as reported in studies).

More extensive data charting was conducted for studies with eligible data for 10 or more participants, including: participants’ age at cochlear implantation, preoperative pure-tone average thresholds and speech perception scores, and postoperative outcomes, such as speech perception outcomes in quiet and in noise, and self-report measures of listening and quality of life. Where multiple postoperative time points of outcome measures were reported, the data for the follow-up closest to 12 months postoperatively was extracted. Where available, data relating to device non-use was also extracted.

In addition to group-level data, individual speech performance outcomes were also extracted when reported in studies. These were charted as a proportion of the population that presented a change in relation to preoperative performance, where change of 0–14 percentage points represented limited change, a decrease greater than zero percentage points was considered a decrease in performance, and change greater than 14 percentage points was considered a clinically significant improvement in performance. These criteria are consistent with those previously reported in cochlear implantation efficacy reports [26].

### Synthesis of results

Evidence was mapped by describing frequencies for year and journal of publication, country of data collection, and study design for all included articles. An identification number (ID) was assigned to each article to facilitate reporting of results. These are provided along with data extracted for each article in the supplemental material (see S1 Table). Studies were classified by their design as per the National Health and Medical Research Council in Australia (NHMRC) levels of evidence [27].

To increase the level of evidence and applicability of the synthesis, only articles with ten or more participants with eligible data were further synthesised. This synthesis encompassed data relating to age at implantation, preoperative pure-tone average thresholds, preoperative speech perception), as well as efficacy (i.e. behavioural measures of speech perception) and effectiveness (i.e. self-reported listening, quality of life, and device non-use) measures of cochlear implantation outcomes.

Speech perception outcomes across studies were summarised according to the onset of hearing loss of individuals included in studies. Because of their specificity, outcomes for individuals with single-sided deafness were also synthesised separately. Only total scores of self-report measures were summarised in this review. When articles only reported on results for each questionnaire subdomain, these were extracted to calculate total scores.

Analysis of extracted data was conducted by averaging summary measures of different studies and weighting these by the number of participants in each individual article to account for different sample sizes across studies. Factors such as study design, however, were not accounted for when examining individual outcomes. Reporting of scoring methods for monosyllabic word tests varied across studies. To decrease the risk of bias in the review, these were only summarised when full word scoring was conducted, as this was the method reported in most studies (as opposed to phoneme scoring). A conservative approach was used to calculate effect size measures [Cohen’s d, 28] only for speech perception outcomes in the implanted ear alone and in the best-aided condition.

## Results

### Selection of sources of evidence

**Fig 1:**
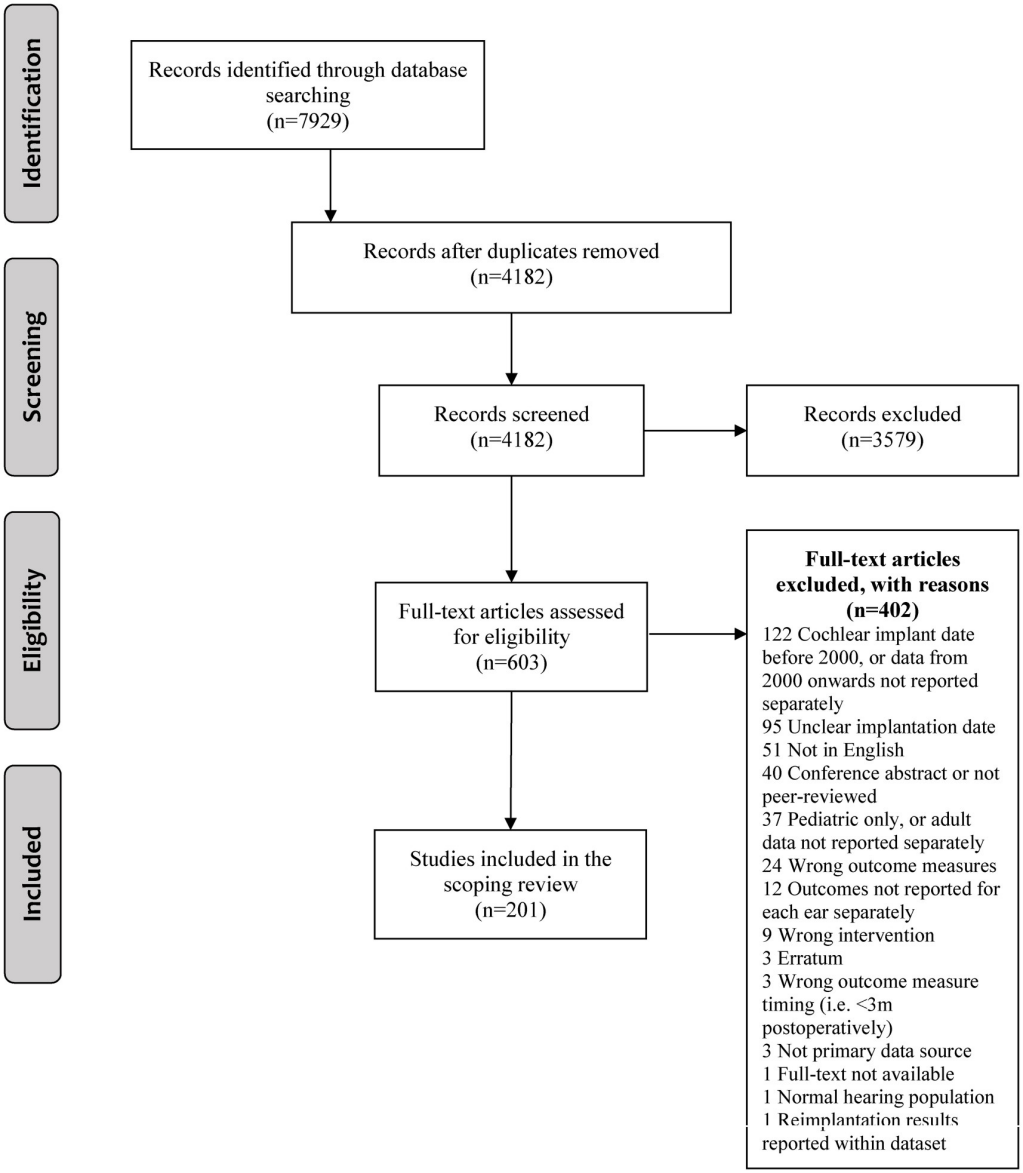
PRISMA flow diagram. *From*: Moher D, Liberati A, Tetzlaff J, Altman DG, The PRISMA Group (2009). *P*referred *R*eporting *I*tems for *S*ystematic Reviews and *Me*ta-*A*nalyses: The PRISMA Statement. PLoS Med 6(7): e1000097. doi:10.1371/journal.pmed1000097 **For more information, visit** www.prisma-statement.org.

The database searches yielded a total of 7929 records. After removal of duplicates, 4182 abstracts were reviewed and the full texts of 603 articles were screened against the inclusion criteria. After the exclusion of 402 articles (Fig 1), 201 articles were included in this scoping review.

### Characteristics of sources of evidence

#### Origin of datasets

**Fig 2:**
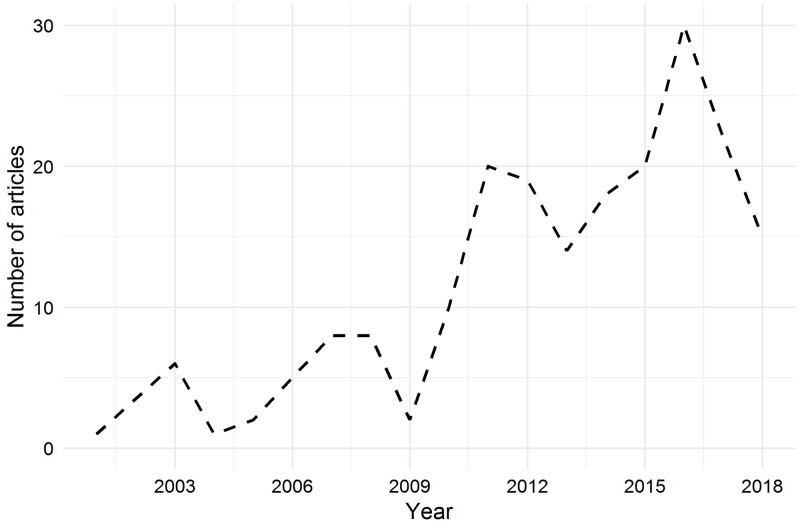
Years of publication of 201 included articles.

**Fig 3:**
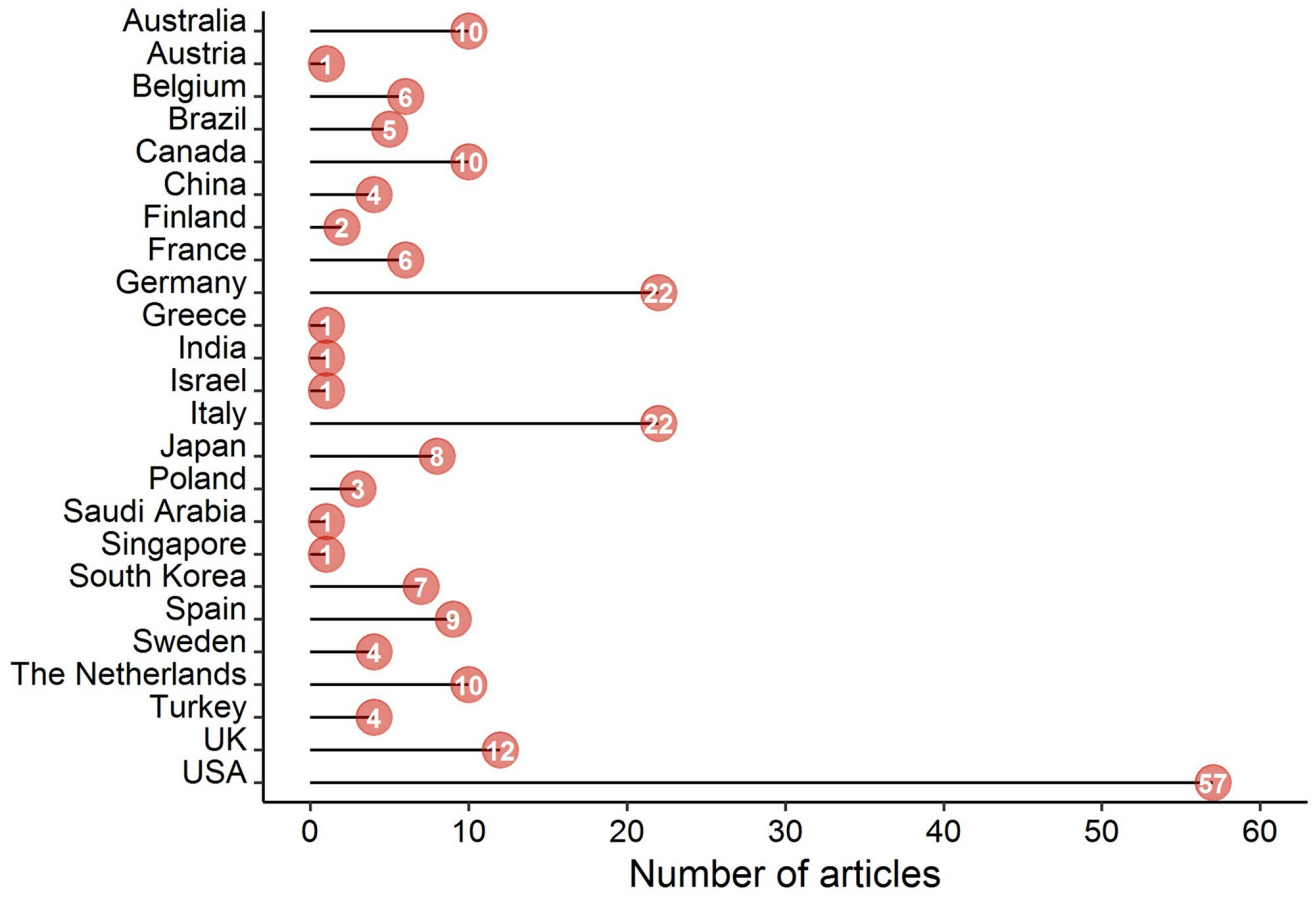
Number of included articles per country.

**Fig 4:**
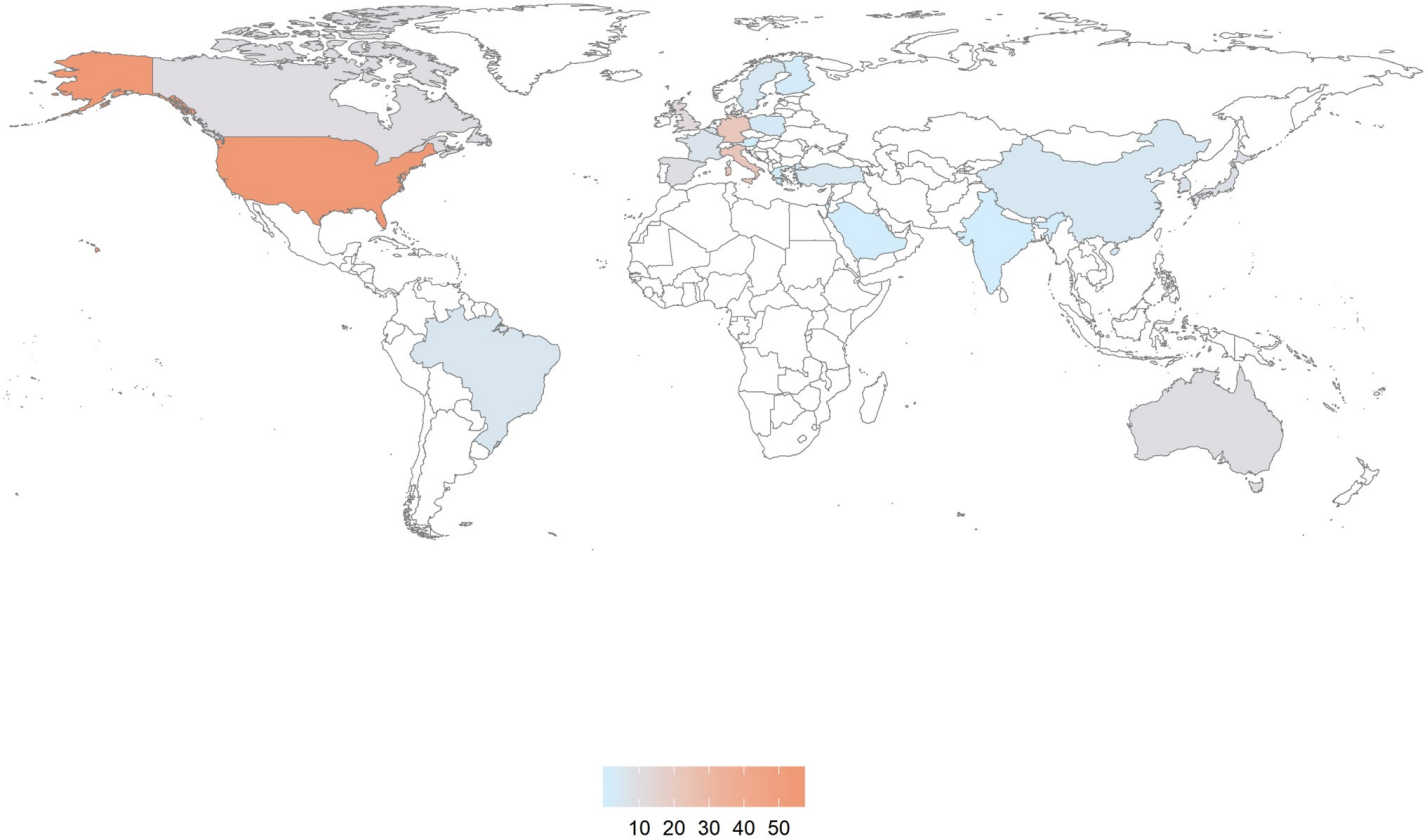
World distribution of countries of data collection for included articles. Please note that Fig 4 was created in R version 3.5.2 [29], using the mapdata package developed by Becker & Wilks for the S software and adapted to R by Brownrigg [30]. This package uses the CIA World Data Bank II (https://www.evl.uic.edu/pape/data/WDB/) public domain data and contains approximately 2 million points representing the world coastlines and national boundaries.

The 201 included articles were published between 2000 and 2018, with the majority of articles published from the year 2010 (Fig 2). Datasets presented in the articles originated from 24 countries, with most originating from the United States (n = 57 articles; 28.4%), followed by Germany and Italy (n = 22 articles each; 10.9%; Figs 3 and 4). When grouped by continents, the majority of articles (46.3%) originated from Europe (Table 1).

**Table 1:** Proportion of articles per continent included in the scoping review (n = 201 articles, including 1 article with dataset collected over 3 continents).

Continent	Articles	Proportion
Americas	72	35.47%
Asia	27	13.30%
Europe	94	46.31%
Oceania	10	4.93%

#### Publication journals

Articles included in this review were published across 45 scientific journals (Table 2). The 5-year impact factor of these journals (a widely used proxy measure of quality) ranged from 0.354 (B-ENT) to 10.840 (Brain). Ten journals did not have impact factors listed in the Journal Citation Reports (Web of Science). Otology and Neurotology was the journal with the most articles included in this review (n = 60), followed by Laryngoscope (n = 16).

**Table 2:** Frequency and journal impact factors of the 201 included articles.

Journal	Impact Factor	N articles	Journal	Impact Factor	N articles
Brain	10.84	1	European Archives of Oto-Rhino-Laryngology	1.546	9
Journal of Neurosurgery	4.318	1	Annals of Otology, Rhinology and Laryngology	1.513	1
Orphanet Journal of Rare Diseases	3.607	1	Auris Nasus Larynx	1.387	4
JAMA Otolaryngology—Head and Neck Surgery	3.295	2	NeuroReport	1.266	3
Ear and Hearing	3.120	12	Acta Otorhinolaryngologica Italica	1.196	2
Frontiers in Human Neuroscience	2.871	1	Acta Oto-Laryngologica	1.161	11
Hearing Research	2.824	3	American Journal of Otolaryngology	1.046	6
PLoS ONE	2.766	2	Orl-Journal for Oto-Rhino-Laryngology Head and Neck Surgery	1.012	1
Clinical Otolaryngology	2.696	1	Journal of Laryngology and Otology	0.967	6
BioMed Research International	2.583	1	Hno	0.893	1
Clinical Interventions in Aging	2.505	2	Journal of International Advanced Otology	0.758	1
Otolaryngology—Head and Neck Surgery	2.444	2	B-Ent	0.354	5
Laryngoscope	2.442	16	Audiology and Neurotology Extra	-	1
Otology & Neurotology	2.182	60	Audiology Research	-	1
Audiology and Neurotology	2.078	9	Case Reports in Ophthalmology	-	1
Trends in Hearing	2.000	2	Cochlear implants international	-	13
Rheumatology International	1.952	1	Current Aging Science	-	1
Medical Science Monitor	1.894	1	Hearing, Balance and Communication	-	1
International Journal of Audiology	1.759	2	Journal of Medical Case Reports	-	1
Journal of Otolaryngology—Head and Neck Surgery	1.704	1	Laryngoscope Investigative Otolaryngology	-	1
BMC surgery	1.692	1	Orl-Journal for Oto-Rhino-Laryngology & its Related Specialties	-	2
Journal of the Acoustical Society of America	1.605	1	Revista Brasileira de Otorrinolaringologia	-	2
Journal of the American Academy of Audiology	1.593	4			

#### Study designs—Levels of evidence

The included articles encompassed evidence levels from III to IV as classified by the NHMRC levels of evidence hierarchy [27]. These consisted mainly of evidence level IV (case series; n = 95), followed by III-3 (comparative studies without a concurrent group; n = 55), and III-2 (comparative study with concurrent group(s); n = 50). No pseudorandomised (III-1) or randomised controlled trials (II) were identified within the included articles. Therefore, no systematic reviews of level II studies (I) were available.

#### Sample sizes

The total number of participants with eligible data ranged from 1–382 participants per study (median: 10). Out of the 201 articles, 99 (49.3%) included less than 10 participants (ID 103–201). The majority of articles with these small sample sizes targeted a specific population characteristic in relation to cochlear implantation outcome, as presented in Table 3.

**Table 3:** Characteristic of populations targeted in articles with small sample sizes (n<10).

Target population characteristic	N participants	N articles
Specific aetiologies	200	71
Comorbidities	32	12
Duration of deafness	4	1
Hearing asymmetry, inc. SSD	53	11
Older age	9	2
Residual hearing preoperatively	22	3
Surgical considerations	8	2

**Fig 5:**
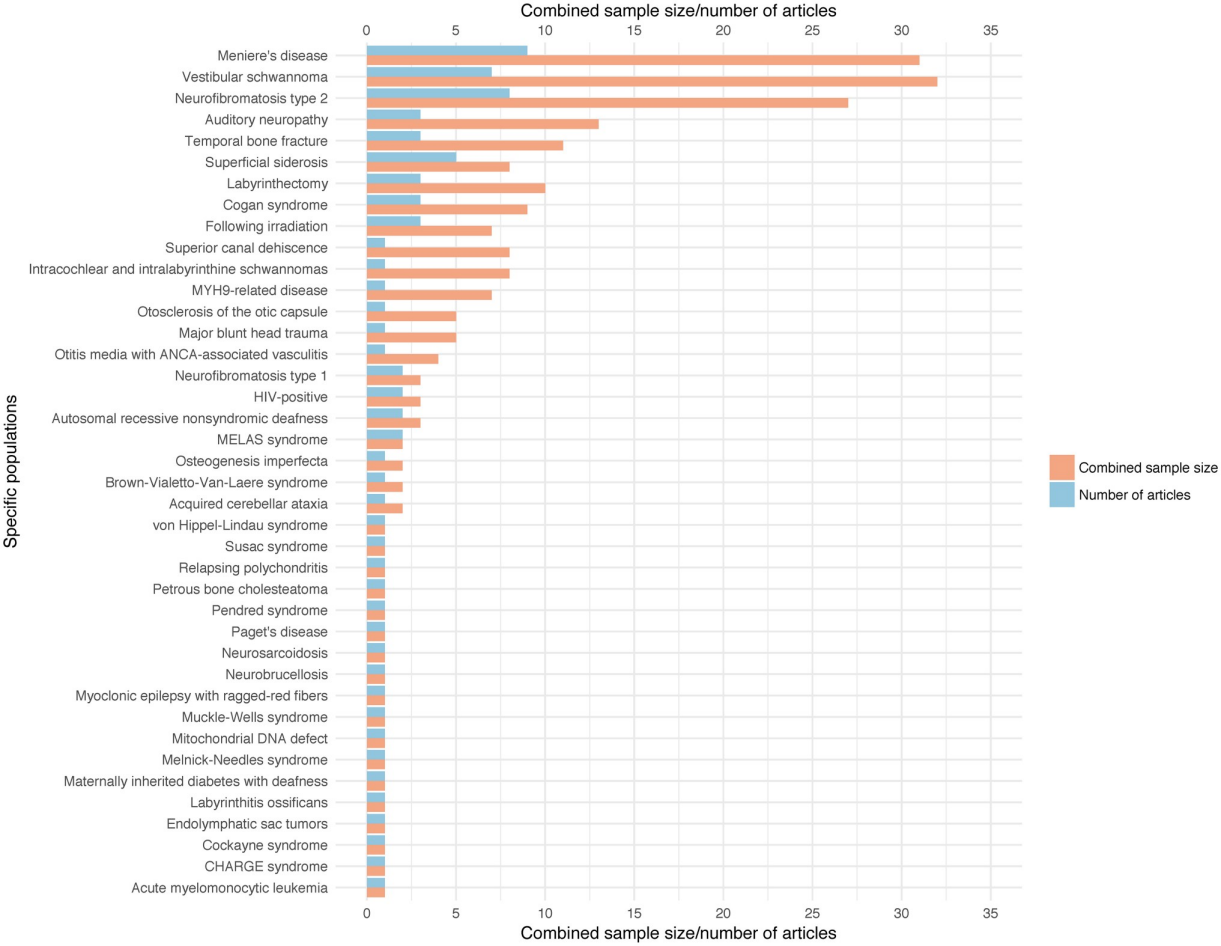
Distribution of aetiologies across articles with small sample sizes (n <10).

The specific etiologies targeted within the small sample size articles are further described in Fig 5. These etiologies mainly related to conditions affecting the inner ear (31 articles), followed by conditions affecting the auditory pathway beyond the cochlea (24 articles), or causing abnormal bony formations (5 articles; see S2 Table for more detail). Because the technology of the cochlear implant device is based on the function of an inner ear with normal structure and tonotopic arrangement of neurons, greater outcome uncertainty is usually expected with aetiologies affecting cochlear structure or more central elements of the auditory pathway.

The proportion of case series (level IV evidence) decreased from 47.5% to 14.9% after excluding articles with n<10, to the benefit of level III evidence. To increase representability of the general population using cochlear implants, as well as confidence in the data synthesis, further extraction and analyses were conducted only with the 102 articles that included at least ten participants (ID 1–102).

#### Synthesis of results

(102 articles with n ≥10 participants).

### Preoperative characteristics

#### Age

Mean age at implantation was found in 95 articles, including 4994 participants. Across articles the mean age at implantation was 61.4 years and the median 63.3 years (range: 18.0–93.4; available from 81 articles). Half the articles included at least one participant implanted after 77 years of age.

#### Onset of hearing loss

**Fig 6:**
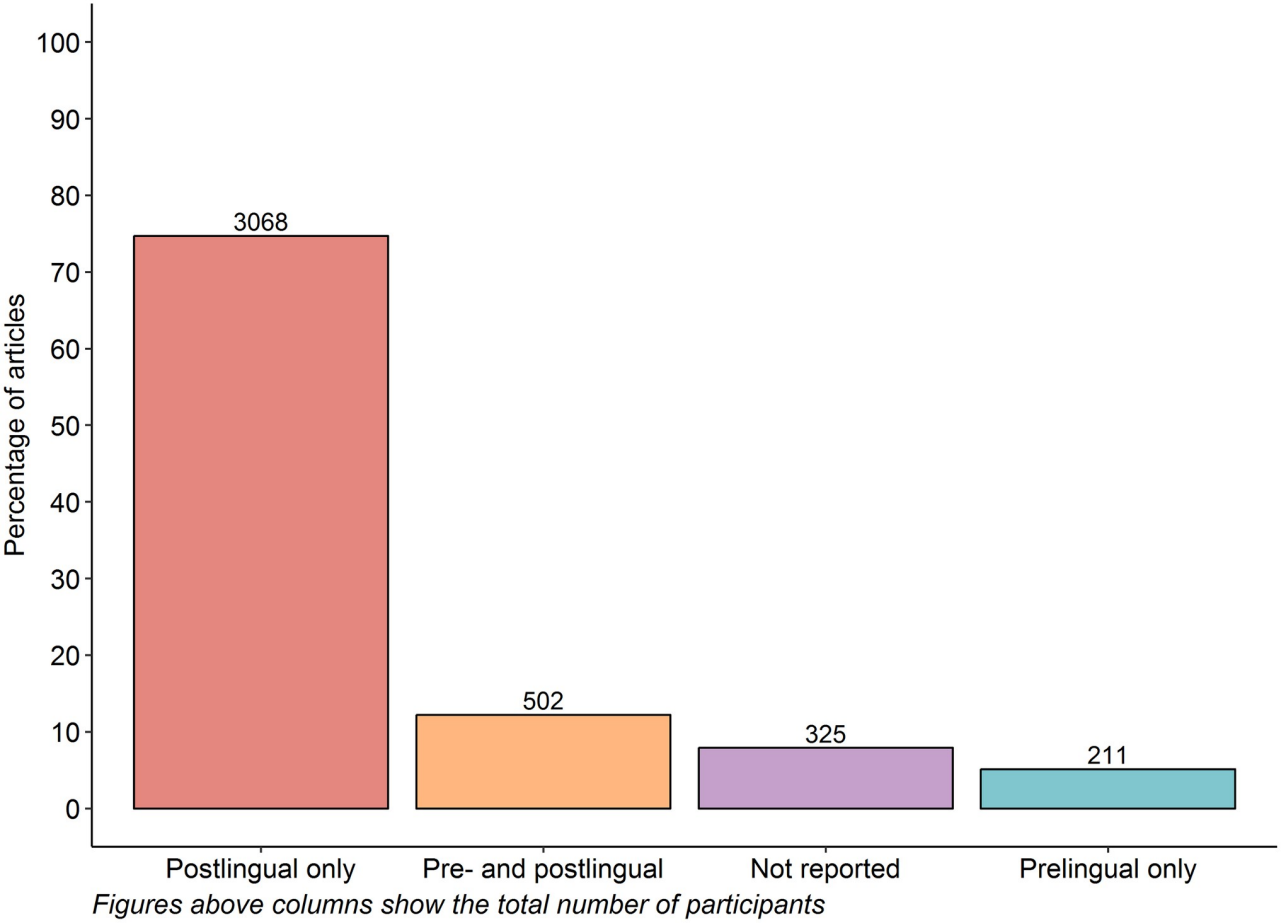
Onset of hearing loss as reported in 102 articles with ten or more eligible participants.

Adults with a postlingual hearing loss constituted at least 74.7% of participants included across all articles, whereas only 5.1% of all participants were represented in articles that reported exclusively on adults with prelingual hearing loss. The onset of hearing loss was not reported for 7.9% of the included articles (Fig 6).

#### Hearing loss severity; ear to be implanted

Preoperative average pure-tone detection thresholds (hearing loss severity) in the ear to be implanted were reported across various combinations of frequencies in 56 articles. The most common combinations reported were a 4-frequency average (.5, 1, 2, 4 kHz; n = 23 articles) and a 3-frequency average (.5, 1, 2; n = 20 articles). Mean and range of hearing loss severity are shown in Table 4. Seven articles included participants with pure-tone threshold averages in the ear to be implanted that would be considered better than a severe hearing loss (3 or 4f PTA <65dBHL). For three of these articles (ID 32, 45, 57), specific aetiology considerations were suggested to justify implantation despite greater residual hearing, i.e. auditory neuropathy, neurofibromatosis type II with vestibular schwannoma, and Meniere’s disease. For one article (ID 59), the implantation candidacy criteria was based on the 25^th^ percentile of speech perception performance of adult cochlear implant users with postlingual hearing loss, independent of hearing loss severity measured with pure-tone thresholds. As such, the article included participants with maximum phoneme perception scores of 46%, sentence perception scores of 88%, and a minimum reported PTA in the ear to be implanted of 57dBHL. The two other articles (ID 5, 79) specifically aimed to assess outcomes in adults with more residual hearing, based on preoperative speech perception performance.

**Table 4:** Mean, standard deviation (SD), minimum and maximum range of pure-tone average (PTA) hearing thresholds reported in the two most common frequency combinations across articles.

Frequency combination		Value	N participants	N articles
3f (.5, 1, 2 kHz)	Mean PTA (dB)	99.71	805	18
	Mean SD (dB)	14.03	595	16
	Min range (dB)	55.0–108.0	298	12
	Max range (dB)	94.4–130.0	298	12
4f (.5, 1, 2, 4 kHz)	Mean PTA (dB)	99.98	765	18
	Mean SD (dB)	15.33	546	14
	Min range (dB)	50.0–91	515	10
	Max range (dB)	97.0–130.0	515	10

#### Hearing loss severity; contralateral ear

Hearing loss severity for the non-implanted contralateral ear ranged from total hearing loss to normal hearing levels. Most articles included individuals who would be considered typical cochlear implant candidates, i.e. participants with a symmetrical severe-to-profound hearing loss bilaterally. Fifteen articles included a total of 345 participants (8.4%) with single-sided deafness (SSD), i.e. participants who had normal hearing or mild to moderate hearing loss in their non-implanted ear.

#### Speech perception ability

Different measures of preoperative aided speech perception ability were available in 72 articles (Table 5). For 44 of these, the measures were available for the ear to be implanted. For 25 articles, the preoperative measures were conducted in the best-aided condition only, which includes bilateral hearing configurations and is generally a better representation of the hearing ability of the better ear rather than the ear to be implanted. In six articles, the listening condition in which preoperative speech perception was measured was not reported (ID 22, 46, 61, 64, 76, 94). Four articles specifically aimed to measure post-implantation outcomes in adults with better speech perception abilities (ID 5, 79, 28, 72). As shown in Table 6, overall, participants had a mean preoperative aided word score of 8.3% and a mean preoperative aided sentence in quiet score of 19.5% in the ear to be implanted. The maximum preoperative aided scores were 62% and 91%, for words and sentences respectively, in the ear to be implanted.

**Table 5:** Distribution of pre-implantation aided speech perception tests reported across articles.

Preoperative measures	Ear to be implanted	Best-aided
	Number of articles	
Monosyllabic words	27	11
Sentences in quiet	14	7
Phonemes in monosyllabic words	6	3
Multisyllabic words	7	1
Sentences in noise	4	3
Other or unspecified	11	2

**Table 6:** Preoperative mean speech perception score, standard deviation (SD), minimum and maximum range, as measured with the most common speech perception tests with the ear to be implanted alone.

**Monosyllabic words in quiet**		**Value**	**N participants**	**N articles**
Presentation levels (dB SPL): 60 (32%), 65 (36%), 70 (7%), ? (18%), live voice (7%)				
	Mean score (%)	8.30	1351	25
	Mean score (SD)	12.39	518	14
	Min range	0–4.0	253	9
	Max range	10.5–62.0	264	10
**Sentences in quiet**		**Value**	**N participants**	**N articles**
Presentation levels (dB SPL): 50 (7%), 60 (29%), 65 (14%),? (36%), live voice (14%)				
	Mean score (%)	19.45	581	13
	Mean score (SD)	13.62	323	11
	Min range	0–49.0	77	7
	Max range	0–91.0	77	7

### Postoperative speech perception outcomes (efficacy)

#### Group-level outcome and change

Many different types of speech perception test were used to report on postoperative outcomes, generally for the implanted ear alone. The most frequently reported speech perception measures used after implantation were monosyllabic words (46 articles), sentences in quiet (34 articles), and sentences in noise (collocated speech and noise in front of the participant [S0N0]; 13 articles; Table 7), for the implanted ear alone. Outcome measures were available at the 12-month post-implantation time point for 48% of articles. When the 12-month measure was not available, the closest to the 12-month time point was selected, which resulted in 1% reporting at 3 months, 16% reporting between 6 and 9 months, 15% reporting at more than 12 months and 20% reporting undefined timing greater than 3, 6, or 9 months post-implantation.

**Table 7:** Postoperative mean speech perception score, standard deviation (SD), minimum and maximum range, as measured on different tests with the cochlear implant (CI) ear alone.

**Monosyllabic words in quiet**		**Value**	**N participants**	**N articles**
Presentation levels (dB SPL): 60 (20%), 65 (37%), 70 (13%), ? (24%), live voice (6%)				
	Mean score (%)	54.00	2798	46
	Mean score (SD)	22.50	1164	27
	Min range	0–46.0	1125	23
	Max range	10.46–100.0	1125	23
	25^th^ percentile	41.97	457	7
	Mean improvement CI alone	48.13	887	16
	Mean improvement best aided	44.37	631	12
**Sentences in quiet**		**Value**	**N participants**	**N articles**
Presentation level (dB SPL): 50 (3%), 60 (18%), 65 (36%), 70 (21%), ? (42%), live voice (9%)				
	Mean score (%)	74.37	1815	34
	Mean score (SD)	36.98	1102	24
	Min range	0–76	773	19
	Max range	7.24–100.0	773	19
	25^th^ percentile	59.98	454	6
	Mean improvement CI alone	54.75	468	10
	Mean improvement best aided	50.49	491	10
**Sentences in noise S0N0**		**Value**	**N participants**	**N articles**
Fixed SNR levels (dB): +5 (21%), +8 (7%), +10 (43%), +15 (14%), ? (14%)				
	Mean score (%)	49.78	454	12
	Mean score (SD)	27.27	336	10
	Min range	0.0–34.0	158	8
	Max range	20.0–100.0	158	8
	25^th^ percentile	4.05	123	2

Two articles (ID 19, 96) presented sentence in noise perception results as a signal-to-noise ratio required to obtain 50% correct with the cochlear implant alone, in the S0N0 noise configuration. It can be assumed that this test is only conducted when patients obtain a minimum score of 50% correct on sentence perception in quiet, and therefore these results would not be representative of the lowest performing quarter (based on a 25^th^ percentile of 60% as calculated from six articles). The more residual hearing present in the contralateral ear, the more likely the outcomes were also reported in the best-aided condition. For the majority of articles that included patients with SSD, sentence perception in noise tests were conducted binaurally, in different configurations of separated speech and noise.

**Fig 7:**
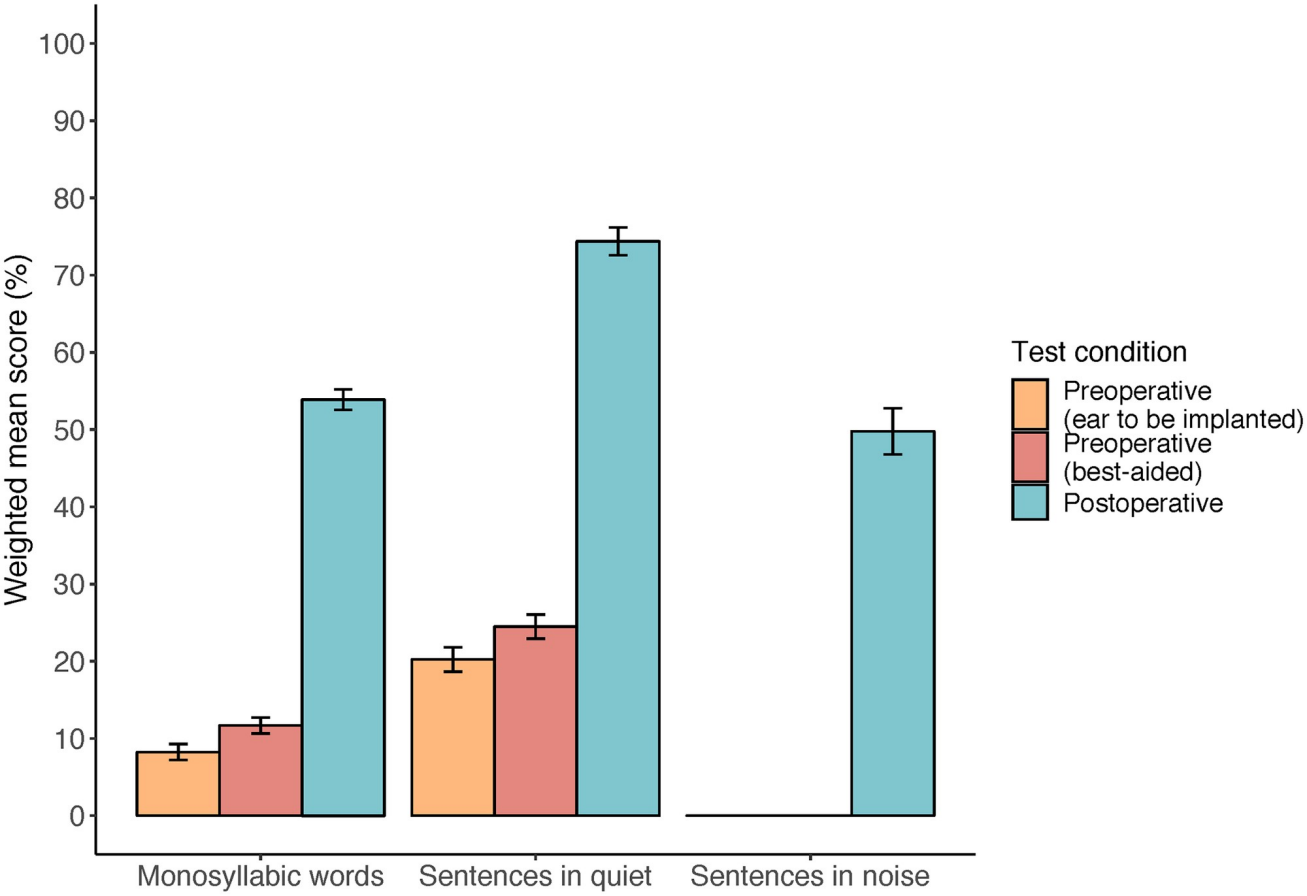
Postoperative speech perception performance in the CI ear alone compared with pre-operative performance for three outcome measures. Error bars represent the 95% confidence interval.

Across articles, the mean postoperative word, sentence in quiet and sentence in noise scores with the cochlear implant alone were 54%, 74% and 50% (Fig 7). Cohen’s d was calculated to estimate the effect size of mean postoperative change for words (cochlear implant alone: 1.75; best-aided: 1.62) and sentences in quiet (cochlear implant alone: 1.37; best-aided: 1.26). Only slight variations in outcomes were found when looking at subsets of participants, such as adults with postlingual or prelingual hearing loss, adults with SSD, older versus younger adults, or adults with better preoperative speech perception scores, although confidence in these results was somewhat limited by the small number of articles with consistent reporting (see S3, S4 and S5 Tables). Adults with prelingual hearing loss appeared to show the greatest difference in performance compared to other subsets of participants, obtaining an average of 27% and 39% on word and sentence in quiet perception postoperatively.

### Postoperative self-reported outcomes (effectiveness)

#### Group-level outcome measure and change

Within the 102 articles with ten or more eligible participants, 36 assessed cochlear implantation effectiveness with formal self-report measures of listening or quality of life. Table 8 describes the 16 outcome measures used across studies and the articles in which these were utilised. Questionnaires also varied in terms of response scale and method of calculating improvement in performance.

**Table 8:** Self-report measures of listening or quality of life identified across included studies.

Outcome measure	Description	N articles [ID]
APHAB	Measures unaided and aided performance as well as hearing aid benefit in relation to speech understanding in different environments and by quantifying negative reactions to environmental sounds. Subdomains: ease of communication, reverberation, background noise, aversiveness.	5 [33, 86, 89, 90, 96]
BBSS	Assesses aided and unaided performance in different listening situations.	2 [35, 87]
CAP	Index consisting of eight auditory performance categories arranged in order of increasing difficulty.	3 [40, 69, 98]
EQ-5D-3L-VAS	Assesses mobility, self-care, usual activities, pain/discomfort and anxiety/depression	1 [60]
GBI	Measures change in health status resulting from health care intervention and was developed especially for otorhinolaryngological interventions.	5 [24, 43, 54, 94, 95]
GHABP	Assesses disability, handicap and hearing aid benefit	1 [60]
HUI2	Measures general health status and health-related quality of life. Subdomains: sensation, mobility, emotion, cognitive, self-care, pain, fertility.	1 [52]
HUI3	Measures general health status and health-related quality of life. Subdomains: Vision, hearing, speech, ambulation, dexterity, emotion, cognition, pain.	6 [6, 64, 84, 85, 90, 102]
HHI	Assesses the emotional and social impact of hearing loss on quality of life.	2 [5, 78]
HHIE	Assesses effects of hearing loss on the emotional and social adjustment of elderly people.	1 [64]
IOI-CI	Assesses cochlear implant usage, benefit, residual activity limitations, residual participation restrictions, satisfaction, impact on others, and quality of life.	1 [6]
NCIQ	Health-related quality of life instrument for use in cochlear implant users. Assesses physical, psychological and social domains. Subdomains: basic sound perception, advanced sound perception, speech production, self-esteem, activity, social interactions.	9 [16, 52, 53, 60, 71, 73, 76, 77, 91]
Oldenburg Inventory	Assesses listening in quiet, listening with background noise, and localisation.	2 [16, 53]
SF-36	Measures health-related quality of life via eight subscales: physical functioning, role-physical, bodily pain, general health, vitality, social functioning, role-emotional, and mental health.	4 [16, 25, 34, 91]
SSQ	Measures hearing disability and handicap by assessing speech hearing, spatial hearing, and qualities of hearing.	8 [6, 33, 36, 37, 60, 82, 83, 96]
WHOQOL-OLD	Assesses sensory abilities; autonomy; past, present and future activities, social participation, death and dying and intimacy in older people.	1 [93]

APHAB, Abbreviated Profile of Hearing Aid Benefit; BBSS, Bern Benefit in Single-Sided Deafness Questionnaire; CAP, Categories of Auditory Performance; EQ-5D-3L-VAS, EQ-5D-3L Visual Analogue Scale; GBI, Glasgow Benefit Inventory; GHABP, Glasgow Hearing Aid Benefit Profile; HUI2, Health Utilities Index 2; HUI3, Health Utilities Index 3; HHI, Hearing Handicap Inventory; HHIE, Hearing Handicap Inventory for the Elderly; IOI-CI, International Outcome Inventory for Cochlear Implants, NCIQ, Nijmegen Cochlear Implant Questionnaire; SF-36, Short Form-36; SSQ, Speech, Spatial and Qualities of Hearing Scale; WHOQOL-OLD, World Health Organisation Quality of Life for older adults.

Most articles containing self-report outcome measures reported on cochlear implantation outcomes exclusively for adults with postlingual hearing loss (91.8%), whereas 2.5% included only adults with prelingual hearing loss. Another 2.5% evaluated both populations together, and 3.3% articles did not report onset of hearing loss for their study participants. Nine articles (25%; ID 6, 33, 35, 60, 82, 83, 87, 89, 96) reported on outcomes of adults with SSD.

**Fig 8:**
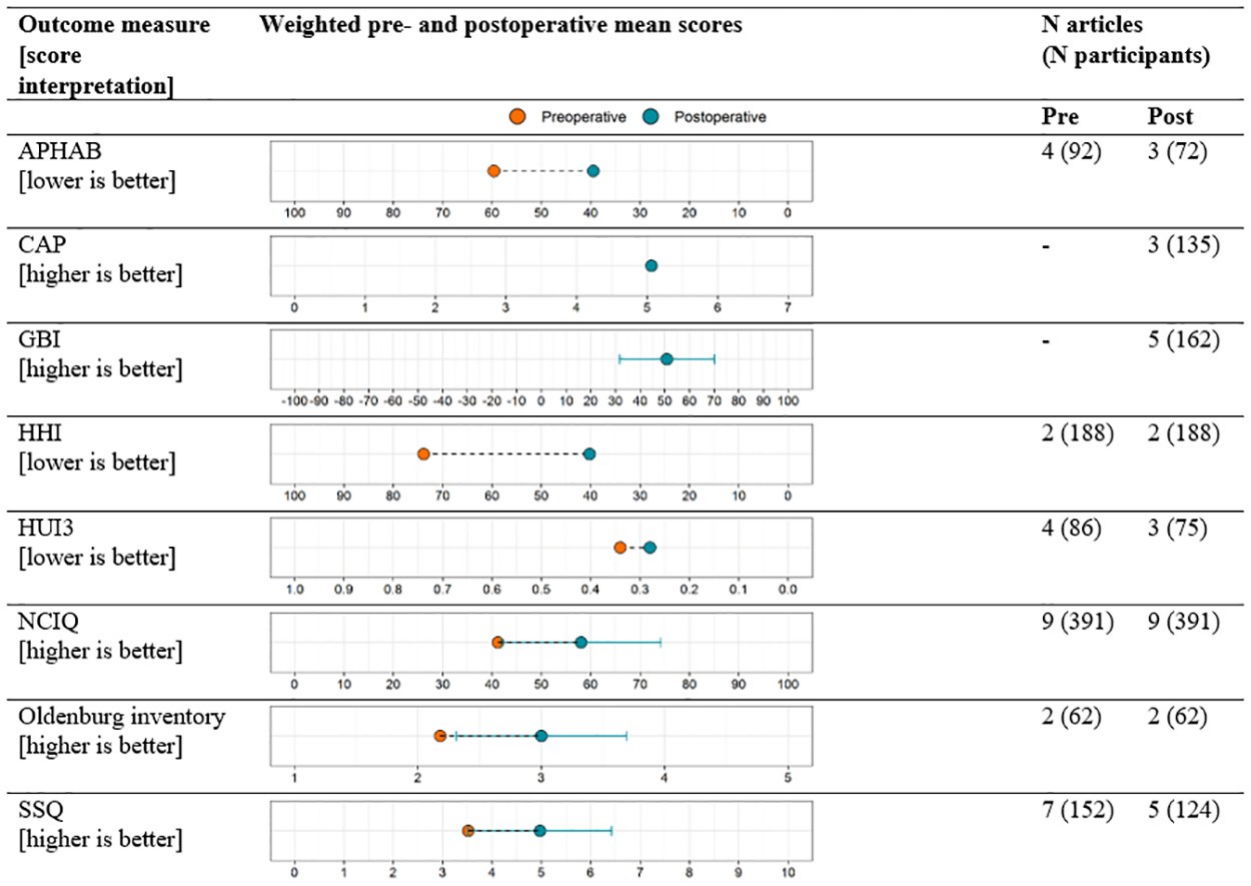
Pooled data from studies reporting on formal self-report outcome measures. APHAB, Abbreviated Profile of Hearing Aid Benefit; CAP, Categories of Auditory Performance; GBI, Glasgow Benefit Inventory; HUI3, Health Utilities Index 3; HHI, Hearing Handicap Inventory; NCIQ, Nijmegen Cochlear Implant Questionnaire; SSQ, Speech, Spatial and Qualities of Hearing Scale. Error bars show weighted standard deviation.

In general, postoperative improvement in self-report measures of listening or quality of life was shown in most articles, except for one (ID 60) that showed no mean postoperative change on the Nijmegen Cochlear Implant Questionnaire in adults with SSD (see S6 Table). Results were pooled for each questionnaire separately (Fig 8) when data were reported as means and standard deviations. Taken together, a weighted average of all available results suggested a mean improvement across 933 participants of 21.5 percentage points.

### Individual change post-implantation (probability of improvement)

**Fig 9:**
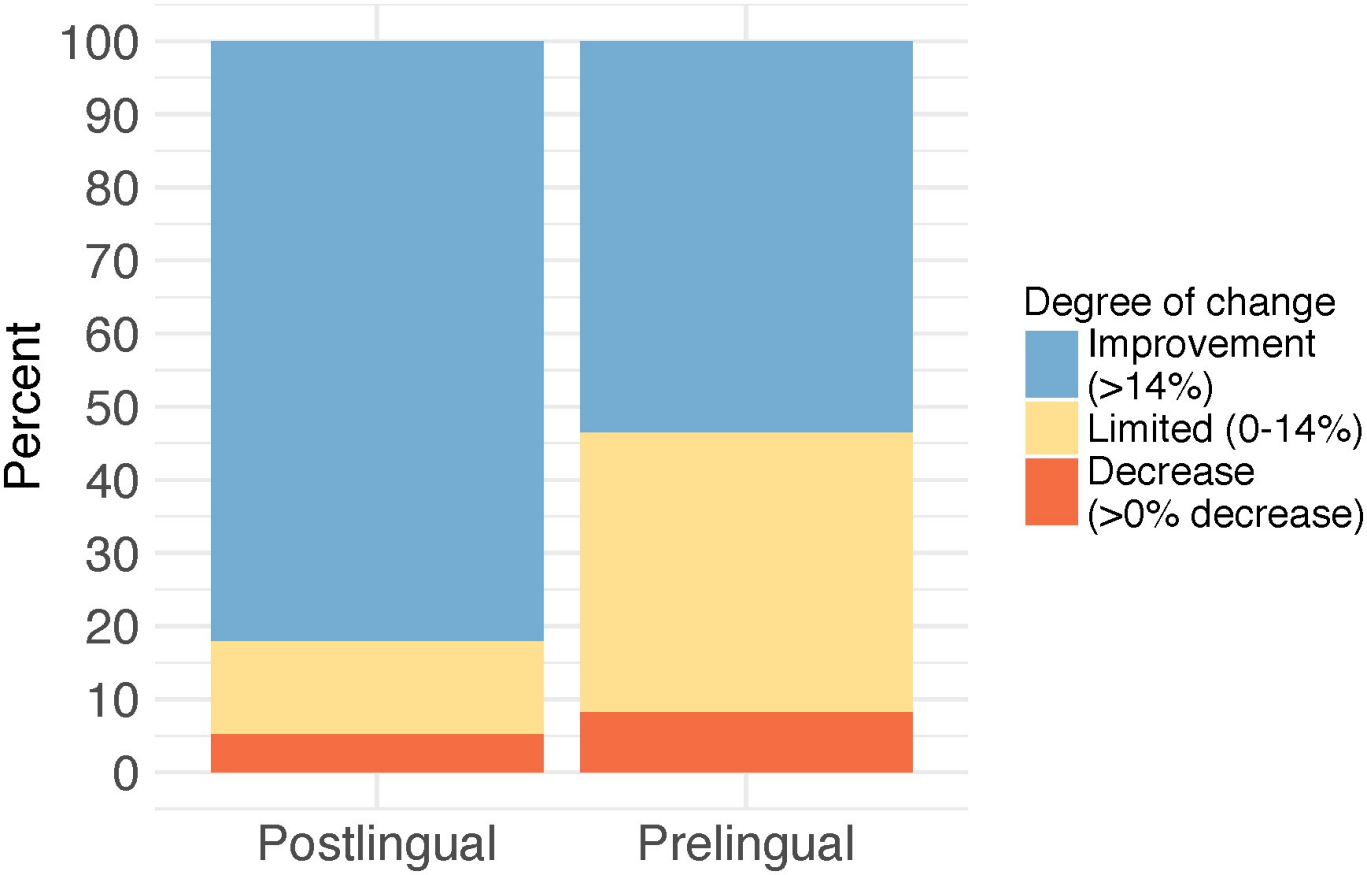
Individual change in speech perception performance in quiet for adults with pre- and postlingual hearing loss.

Fourteen articles reported on individual change post cochlear implantation. A typical population of adults with postlingual bilateral hearing loss (excluding articles focusing on SSD, prelingual hearing loss, and vestibular schwannoma) was represented in five articles (480 participants; ID 37, 69, 72, 92, 101). 82.0% of this population showed a greater than 14 percentage point improvement in speech perception scores in quiet following cochlear implantation (Fig 9). Limited change (0–14 percentage points) was shown for 12.7% of the population, while a decrease in speech perception performance post-implantation (>0% decrease) was shown for 5.3% of the population. A similar pattern of individual change was found for speech perception in noise, as presented in two articles (53 participants; ID 72, 92): 83.0% improved, 13.2% maintained similar scores, and 3.7% obtained a decrease in speech perception in noise performance. Individual change in self-reported outcomes was presented in two articles (128 participants; ID 37, 69), with 91% of these participants improving on self-reported measures post-implantation. It was not possible to calculate the overall proportion of the population that decreased or obtained similar scores on self-reported measures.

Four articles that presented individual change focused specifically on adults with prelingual hearing loss (138 participants; ID 16, 27, 3, 93). The proportion of adults who improved >14 percentage points was smaller in this population compared with adults with postlingual hearing loss. Specifically, for speech perception performance in quiet, 53.4% improved, 38.3% maintained similar scores, and 8.3% obtained poorer scores post-implantation. Speech perception in noise was reported for a subgroup of 19 participants with prelingual hearing loss (ID 29, 36), showing an improvement in 52.6%, similar scores in 42.1%, and a decrease in performance in 5.3% of this group. For adults with prelingual hearing loss, individual change in self-reported measures was only available for 1 study (3 participants) and is therefore not reported here.

### Device use/ non-use

Twenty articles reported on device use post-implantation, including 992 participants. Non-users were reported in 10 of these articles (n = 30 participants; 3.0%). More than half of the non-users were adults with prelingual hearing loss (n = 16; 53.3%), followed by adults with neurofibromatosis type II (n = 8; 26.7%).

## Discussion

This scoping review aimed to appraise and integrate recent evidence of cochlear implantation outcomes in adults. This knowledge was gathered to support the further development of consistent and clinically aligned candidacy criteria, as well as the design and reporting of future clinical studies.

After a thorough database search and eligibility assessment, 201 articles were initially included in the review. While this is a large amount of recent evidence regarding cochlear implantation outcomes in adults, this evidence is mainly based on case-series study designs (NHMRC level IV) or small sample sizes (<10 participants). No randomised controlled trials have been published on the efficacy of cochlear implantation in adults with severe-to-profound hearing loss, without a predefined selection criteria using speech perception with hearing aids. Current knowledge about cochlear implantation efficacy is therefore derived from the postoperative performance of adults who have most likely received a cochlear implant because of poor aided hearing, as determined by clinics who may be applying inconsistent criteria [16]. In addition, the available evidence is often inconsistently reported across a range of scientific publications with different reviewing standards. Considerable variability was found in reporting core data, the material used for testing, the presentation of this material, the timing of post-implant assessments, and the listening conditions in which the participants were tested. While the types of speech perception tests vary across studies, there appears to be some consensus on the use of monosyllabic word tests (scored as full words rather than phonemes) presented at 65 dB SPL. Usage rate, pre- and postoperative outcome data were extracted from the 102 articles that included 10 or more eligible participants.

The majority of adults included across these articles were aged above 60 years old. The majority also had an acquired (postlingual) hearing loss, which has been demonstrated to lead to significantly better outcomes in comparison to early onset (prelingual) hearing loss. On average, included participants had very poor speech perception performance with their hearing aids before receiving a cochlear implant. This average level of pre-implant performance was considerably below the range of current criteria used across different countries for recommending a cochlear implant [c.f. 16]. This suggests that for the majority of these adults, cochlear implantation is typically delayed many years beyond the time they could potentially have gained benefit from the device. On the other hand, adults with a wide range of hearing thresholds and speech perception performances were also included. The results confirm that audiological candidacy criteria have been relaxed over the years to include adults with less than severe hearing threshold levels, to the extent of any hearing threshold level in one or both ears, if poor speech perception results are obtained with hearing aids. Overall, pre-implantation speech perception results appeared to be the main audiological characteristic considered when determining candidacy, and therefore study eligibility, in the articles reviewed. This contrasts with the general suggestion that cochlear implants are recommended devices for adults with severe-to-profound hearing loss. It should be noted that this scoping review also included articles that investigated outcomes of cochlear implantation in populations with characteristics that are beyond current indications approved by the American Food and Drug Administration [c.f. 8].

### Usage rate

Based on the available articles that reported on device use, a very high majority of adults (97%) who undergo cochlear implantation continue to utilise the device consistently in their everyday life. This usage rate is similar to an earlier report with participants with more severe hearing losses that found a usage rate of 98% within one cohort of 313 postlingually deafened adults in the UK [31].

### Speech perception improvement (efficacy)

Two pre/post comparisons of speech perception performance were mainly found in the included articles: the difference between word perception scores for the ear implanted, and the difference in the “best-aided” condition that will often involve the binaural use of conventional hearing aids during the pre-implant assessment. Across the articles, group-level word perception scores improved from 8.2% pre-implant to 53.9% post-implant for the implanted side, corresponding to a large effect size. This confirms the overall efficacy of the intervention, but also the known limitations of cochlear implantation in restoring a perception of normal hearing. Furthermore, a majority of adults (82%) with postlingual hearing loss obtained an individual speech perception improvement of 15 percentage point or greater in the implanted ear. This proportion was smaller for adults with prelingual hearing loss (53.4%). Articles reporting pre-and post-implant scores for the best-aided condition indicated a mean improvement of 44.4%. These results also implied that recipients with very poor speech perception before implantation have a high probability of improvement. This probability—and the magnitude of improvement—decreases with better functional hearing prior to implantation. For instance, a number of studies included in this review investigated cochlear implantation for single-sided deafness, where the non-implanted ear has effectively normal hearing and speech perception. While efficacy has been demonstrated with improvement in speech perception in the implanted ear [32, 33], the probability of overall improvement when listening with both ears with standard word perception tests in quiet is zero in such cases, as the normal ear would score 100%. More sophisticated testing has, however, demonstrated enhancement of speech perception in noisy environments for some of these recipients [32, 34]. The probability of functional improvement after cochlear implantation also decreases with more hearing in the ear to be implanted, particularly as the pre-implant word perception score for this ear approaches the mean post-operative performance with a cochlear implant [35]. Despite improvement in hearing preservation techniques during implantation, the surgery is likely to reduce or eliminate any residual hearing in the ear to be implanted [36–38]. When there is significant useful speech perception in either ear, careful consideration is needed on an individual basis with regard to probability of benefit, and risk of losing residual hearing in the implanted ear.

### Self-reported benefit (effectiveness)

A large range of questionnaires mainly focusing on hearing and communication benefits have been used to assess quality of life and functional hearing in adult cochlear implant recipients. This makes it difficult to integrate these outcomes in a concise way. In this scoping review, a weighted average of the available self-reported benefit measures confirmed the effectiveness of the intervention across recently implanted adult populations, with a mean improvement across 933 recipients of 21.5 percentage points. A more modest 12 percentage point improvement is seen for the studies utilising a general quality of life measure (HUI3). Nonetheless, consistent self-reported functional benefit is well-demonstrated across the adult cochlear implant population, despite the relaxation of candidacy criteria as shown in this review.

### Factors affecting outcomes

The articles in this review included adults of all ages, with pre and postlingual hearing loss, bilateral and single-sided deafness, and a range of residual hearing abilities in the implanted ear. The results highlight a considerable effect of onset of deafness on outcomes as measured by speech perception tests. Adults with severe bilateral hearing loss from birth or early childhood (prelingual deafness) show significantly poorer implant scores and pre/post improvement than those with acquired (postlingual) deafness [39, 40]. While advanced age [41, 42] and less residual hearing [4] have been associated with poorer speech perception scores in some studies, neither of these factors have been shown to have strong predictive power. This is consistent with the subgroup results of this scoping review (see supplementary file for detail). Studies looking at aetiology of hearing loss generally have found no significant effects, although it is generally accepted that rare cases where the auditory nerve is damaged or the cochlear anatomy is substantially altered may limit benefit from a cochlear implant or prevent successful surgical placement of the device [43]. Consistent with these factors, for the 3.02% of participants reported as device non-users in this review, more than half were adults with prelingual hearing loss (53.33%), followed by adults with neurofibromatosis type II (26.67%).

### Limitations of this scoping review

A formal quality appraisal of the included articles was beyond the scope of the current review. To decrease the risk of bias, this review analysed cochlear implant usage, effectiveness, and efficacy in articles that included at least 10 eligible participants. This scoping review also did not consider complications of cochlear implant surgery, although available reports suggest an eventual surgical success exceeding 98% [44–46]. A potential bias that may be present across multiple articles is the absence of reporting on the number of participants who could not be tested with standard speech perception tests. This population is likely to be over-represented by adults with poor speech perception performance after implantation.

## Conclusions and recommendations

This scoping review provides an integrated descriptive analysis of the current published evidence base for cochlear implantation in adults, yielding the following recommendations:

1. **Cochlear implantation can be considered an effective treatment of hearing loss for adults of any age who have limited speech perception**. Limited speech perception is currently measured with speech perception tests in the ear to-be-implanted, using the best alternative treatment, typically, an acoustic hearing aid. Based on this scoping review, 75% of adults (25^th^ percentile) using a cochlear implant obtained ≥42% on word perception and ≥60% on sentence perception in quiet. Surgical considerations and the integrity of the auditory pathway beyond the cochlea need to be addressed carefully for individual cases.
2. Patients and clinicians can expect that after implantation:
  - **Approximately 82% of patients with postlingual hearing loss, and 53% of patients with prelingual hearing loss will have improved speech perception** performance by 15 or more percentage points in the implanted ear.
  - Very few patients (5–8%) will obtain poorer speech perception with their cochlear implant in comparison to their preoperative performance, but a number may experience no significant change or limited improvement.
  - The greater the preoperative speech perception performance, the smaller the postoperative change. On average, adults obtain a 50 percentage point improvement in their performance.
  - Having a prelingual hearing loss or neurofibromatosis type II/vestibular schwannoma increases the probability of becoming a device non-user.
3. The development of local candidacy criteria should consider the available resources for the population and the consequences of non-treatment. While cochlear implants have been shown to be effective for adults with more residual hearing (including SSD), the largest benefits of cochlear implantation are found with bilateral hearing loss, when speech perception is poor for both ears. **Cost-effectiveness analyses can support decisions related to resource allocation**.
4. While cochlear implants are highly effective in most cases, there is significant variability in outcomes, and some adults will not obtain benefit from them. As such, **complementary or alternative interventions may be required** even after implantation to support effective communication for all adults with hearing loss (for example: captions, adapted acoustic environments, or sign language).
5. To further increase the knowledge base, **minimum reporting standards** need to be considered:
  - Increase standardisation of test material and conditions. From this scoping review, recorded monosyllabic words (scored at the full word level), sentences in quiet, and sentences in noise (+10dB SNR), presented at a level of 65dB SPL (60dBA) from a single loudspeaker in front of the participant were the most commonly used tests. This is aligned with recommendations proposed by Adunka et al. [47].
  - With a considerable increase in the number of adult cochlear implant users having useful hearing in the non-implanted ear (up to normal hearing), standards for monaural and binaural testing also need consideration.
  - Current evidence may be somewhat biased towards high performers who are more likely to be tested with standardised material and included in research studies. To limit this bias, the minimum test battery should be conducted with all patients. In addition, authors should report when participants are excluded because of missing outcome data, and indicate whether the tests used in the study are systematically used with all patients. For example, adaptive speech in noise tests can only be conducted with patients that obtain scores above 50% in quiet, excluding poorer performers from analyses.
  - Individual change in performance should be reported in addition to group data. This recommendation described by Gurgel et al. [48] is also applicable for adults with cochlear implants.
6. Further initiatives that can **strengthen the knowledge base**:
  - Limit small sample size studies to specific conditions that are rare and understudied.
  - Conduct meta-analysis of data from existing small sample size studies, for example combining specific aetiologies, to increase reliability of conclusions.
  - Conduct meta-analysis of predictive factors studies to increase prognostic precision.

## Supporting information

S1 Data Checklist. PRISMA-ScR.
(DOCX)

S2 Data Search strategies. (DOCX)

S1 Table Charted data. (XLSX)

S2 Table Aetiologies. Specific aetiologies targeted in articles with small sample sizes grouped by hearing loss categories.
(DOCX)

S3 Table Words scores CI alone. Word perception scores across different subgroups.
(DOCX)

S4 Table Sentences in quiet scores CI alone. Sentence perception in quiet scores across different subgroups.
(DOCX)

S5 Table Sentences in noise scores CI alone. Sentence perception in noise scores across different subgroups.
(DOCX)

S6 Table Self-reported results. Postoperative improvement in self-report outcome measures and change in relation to preoperative performance.
(DOCX)
