# Supplementary material for: Cochlear implantation outcomes in adults: A scoping review
Source: PLoS One. 2020 May 5;15(5):e0232421. doi: 10.1371/journal.pone.0232421 (PMC7199932; doi:10.1371/journal.pone.0232421)
Supplement: S2 Data — (DOCX) [file pone.0232421.s002.docx]

**S2 Search strategies per database**

***A. Ovid MEDLINE n=1524, 02/08/2018***

Advanced search

Database: **Ovid MEDLINE(R) and Epub Ahead of Print, In-Process & Other Non-Indexed Citations, and Daily**1946 to July 31, 2018

1. (cochlear implant* or cochlear prosth* or hearing prosth* or hearing implant* or aud* prosth* or aud* implant*).mp. (14675)
2. (outcome* or predic* or progn* or factor* or indicat*).mp. (9452584)
3. 1 and 2 (7514)
4. limit 3 to "all adult (19 plus years)" (3211)
5. limit 4 to (english language and yr="2000 -Current") (2554)
6. (adult* not (child* or pediat* or paediat*)).mp. (4516868)
7. 5 and 6 (1524)

***B. Ovid EMBASE, n=1658, 23/08/2018***

Advanced search

Database: **Embase** 1974 to 2018 Week 31

1. (cochlear implant* or cochlear prosth* or hearing prosth* or hearing implant* or aud* prosth* or aud* implant*).mp. (15283)
2. (outcome* or predic* or progn* or factor* or indicat*).mp. (10770302)
3. (adult* not (child* or pediat* or paediat*)).mp. (5764007)
4. 1 and 2 and 3 (2068)
5. limit 4 to (english and yr="2000 -Current" and (adult <18 to 64 years> or aged <65+ years>)) (1658)

***C. Web of Science n=3703, 07/08/2018***

Advanced search, all databases

Timespan=2000–2018
Search language=English

1. TI=("cochlear implant*" OR "cochlear prosthes*" OR "auditory implant*" OR "auditory prosthes*" OR "hearing implant*" OR "hearing prosthes*") OR TS=("cochlear implant*" OR "cochlear prosthes*" OR "auditory implant*" OR "auditory prosthes*" OR "hearing implant*" OR "hearing prosthes*") (18,331)
2. TI=(outcome* or predic* or progn* or factor* or indicat*) OR TS=(outcome* or predic* or progn* or factor* or indicat*) (13,690,115)
3. TS=adult* (4,163,771)
4. #1 AND #2 AND #3 (3,703)

***D. Google Scholar, n=16,900, 13/08/2018* (first 200 hits)**

(cochlea|cochlear|hearing|auditory) (implant|implants|prosthesis|prostheses|prosthetic) (outcome|outcomes|predictor|predictors|predictive|prediction|factor|factors|prognosis|prognostic|indicator|indicators) (adult -child -children -pediatric -paediatric) (adult -child -children -pediatric -paediatric)

Custom range: 2000 – 2018
