# Supplementary material for: Cochlear implantation outcomes in adults: A scoping review
Source: PLoS One. 2020 May 5;15(5):e0232421. doi: 10.1371/journal.pone.0232421 (PMC7199932; doi:10.1371/journal.pone.0232421)
Supplement: S2 Table — Specific aetiologies targeted in articles with small sample sizes grouped by hearing loss categories. (DOCX) [file pone.0232421.s004.docx]

**S4 Table**

**Specific aetiologies targeted in articles with small sample sizes grouped by hearing loss categories**

| Category | Population/aetiology | Number of studies | Combined sample size |
| --- | --- | --- | --- |
| Abnormal bony formations | Acute myelomonocytic leukemia | 1 | 1 |
|  | Labyrinthitis ossificans | 1 | 1 |
|  | Melnick-Needles syndrome | 1 | 1 |
|  | Osteogenesis imperfecta | 1 | 2 |
|  | Otosclerosis of the otic capsule | 1 | 5 |
|  | **Total** | **5** | **10** |
|  |  |  |  |
| Inner ear | Autosomal recessive nonsyndromic deafness | 2 | 3 |
|  | CHARGE syndrome | 1 | 1 |
|  | Endolymphatic sac tumors | 1 | 1 |
|  | Following irradiation | 3 | 7 |
|  | Intracochlear and intralabyrinthine schwannomas | 1 | 8 |
|  | Labyrinthectomy | 3 | 10 |
|  | Major blunt head trauma | 1 | 5 |
|  | Maternally inherited diabetes with deafness | 1 | 1 |
|  | MELAS syndrome | 2 | 2 |
|  | Mitochondrial DNA defect | 1 | 1 |
|  | MYH9-related disease | 1 | 7 |
|  | Myoclonic epilepsy with ragged-red fibers | 1 | 1 |
|  | Neurobrucellosis | 1 | 1 |
|  | Neurofibromatosis type 1 | 2 | 3 |
|  | Otitis media with ANCA-associated vasculitis | 1 | 4 |
|  | Paget's disease | 1 | 1 |
|  | Pendred syndrome | 1 | 1 |
|  | Petrous bone cholesteatoma | 1 | 1 |
|  | Superior canal dehiscence | 1 | 8 |
|  | Susac syndrome | 1 | 1 |
|  | Temporal bone fracture | 3 | 11 |
|  | von Hippel-Lindau syndrome | 1 | 1 |
|  | **Total** | **31** | **79** |
|  |  |  |  |
| Retrocochlear | Acquired cerebellar ataxia | 1 | 2 |
|  | Cockayne syndrome | 1 | 1 |
|  | Brown-Vialetto-Van-Laere syndrome | 1 | 2 |
|  | Neurofibromatosis type 2 | 8 | 27 |
|  | Neurosarcoidosis | 1 | 1 |
|  | Superficial siderosis | 5 | 8 |
|  | Vestibular schwannoma | 7 | 32 |
|  | **Total** | **24** | **73** |
|  |  |  |  |
| Other | Auditory neuropathy | 3 | 13 |
|  | Cogan syndrome | 3 | 9 |
|  | HIV-positive | 2 | 3 |
|  | Meniere's disease | 9 | 31 |
|  | Muckle-Wells syndrome | 1 | 1 |
|  | Relapsing polychondritis | 1 | 1 |
|  | **Total** | **19** | **58** |

*Muckle-Wells syndrome* = Urticarial vasculitis

*Acquired cerebellar ataxia* = Following cerebellar surgery

*Following irradiation* = Irradiated nasopharyngeal carcinoma, irradiated tympanojugular paraganglioma, medulloblastoma treatment in childhood

*Autosomal recessive nonsyndromic deafness* = TMPRSS3 and LOXHD1 mutations
