# Supplementary material for: Cochlear implantation outcomes in adults: A scoping review
Source: PLoS One. 2020 May 5;15(5):e0232421. doi: 10.1371/journal.pone.0232421 (PMC7199932; doi:10.1371/journal.pone.0232421)
Supplement: S3 Table — Word perception scores across different subgroups. (DOCX) [file pone.0232421.s005.docx]

**S5 Table. Word perception scores, CI alone.**

Postoperative monosyllabic perception scores with the implanted ear alone, across different subgroups.

| All populations |  | Value | N participants | N articles |
| --- | --- | --- | --- | --- |
|  | Mean score (%) | 53.87 | 2803 | 46 |
|  | Mean score (SD) | 22.97 | 1207 | 28 |
|  | Min range | 0- 46.0 | 1125 | 23 |
|  | Max range | 10.46 - 100.0 | 1125 | 23 |
|  | 25^th^ percentile | 41.97 | 457 | 7 |
|  | Mean improvement CI alone | 47.81 | 892 | 16 |
|  | Mean improvement best aided | 44.37 | 631 | 12 |
|  |  |  |  |  |
| Postlingual only |  | Value | N participants | N articles |
|  | Mean score (%) | 54.04 | 2232 | 35 |
|  | Mean score (SD) | 23.68 | 810 | 19 |
|  | Min range | 0- 46.0 | 916 | 16 |
|  | Max range | 29.0 – 100.0 | 916 | 16 |
|  | 25^th^ percentile | 41.97 | 457 | 7 |
|  | Mean improvement CI alone | 47.53 | 799 | 14 |
|  | Mean improvement best aided | 42.80 | 404 | 9 |
|  |  |  |  |  |
| Prelingual only |  | Value | N participants | N articles |
|  | Mean score (%) | 26.90 | 54 | 3 |
|  | Mean score (SD) | 21.13 | 38 | 2 |
|  | Insufficient number of articles to combine further measures | | | |
|  |  |  |  |  |
| Postlingual younger adults (<76 year old), no SSD |  | Value | N participants | N articles |
|  | Mean score (%) | 60.57 | 104 | 4 |
|  | Mean score (SD) | 26.13 | 74 | 2 |
|  | Insufficient number of articles to combine further measures | | | |
|  |  |  |  |  |
| Postlingual older adults ( >59 year old; range 60-93 year old), no SSD |  | Value | N participants | N articles |
|  | Mean score (%) | 53.55 | 136 | 4 |
|  | Mean score (SD) | 18.1 | 25 | 1 |
|  | Insufficient number of articles to combine further measures | | | |
|  |  |  |  |  |
| Adults with SSD |  | Value | N participants | N articles |
|  | Mean score (%) | 45.42 | 133 | 5 |
|  | Mean score (SD) | 23.48 | 40 | 3 |
|  | Min range | 0- 10.0 | 60 | 4 |
|  | Max range | 64 - 84 | 60 | 4 |
|  | 25^th^ percentile | 37.34 | 93 | 2 |
|  | Mean improvement CI alone | 51.0 | 20 | 1 |
|  | Mean improvement best aided | na | na | na |
|  |  |  |  |  |
|  |  |  |  |  |
| Adults with more residual hearing pre-CI |  | Value | N participants | N articles |
|  | Mean score (%) | 56.55 | 104 | 3 |
|  | Mean score (SD) | 6.51 | 92 | 2 |
|  | Min range | - 1. 88 | 92 | 2 |
|  | Max range | 22- 95.11 | 92 | 2 |
|  | Mean improvement CI alone | 32.64 | 12 | 1 |
|  | Mean improvement best aided | 37.11 | 27 | 1 |
|  |  |  |  |  |
