# Supplementary material for: Cochlear implantation outcomes in adults: A scoping review
Source: PLoS One. 2020 May 5;15(5):e0232421. doi: 10.1371/journal.pone.0232421 (PMC7199932; doi:10.1371/journal.pone.0232421)
Supplement: S4 Table — Sentence perception in quiet scores across different subgroups. (DOCX) [file pone.0232421.s006.docx]

**S6 Table. Sentence perception in quiet scores, CI alone.**

Postoperative sentence perception in quiet scores with the implanted ear alone, across different subgroups.

| All populations |  | Value | N participants | N articles |
| --- | --- | --- | --- | --- |
|  | Mean score (%) | 74.37 | 1815 | 34 |
|  | Mean score (SD) | 36.98 | 1102 | 24 |
|  | Min range | 0- 76 | 773 | 19 |
|  | Max range | 7.24- 100.0 | 773 | 19 |
|  | 25^th^ percentile | 59.98 | 454 | 6 |
|  | Mean improvement CI alone | 54.75 | 468 | 10 |
|  | Mean improvement best aided | 50.49 | 491 | 10 |
|  |  |  |  |  |
| Postlingual only |  | Value | N participants | N articles |
|  | Mean score (%) | 73.81 | 1366 | 22 |
|  | Mean score (SD) | 21.36 | 769 | 14 |
|  | Min range | 0- 76 | 537 | 9 |
|  | Max range | 93.26- 100.0 | 537 | 9 |
|  | 25^th^ percentile | 59.98 | 454 | 6 |
|  | Mean improvement CI alone | 51.97 | 509 | 8 |
|  | Mean improvement best aided | 51.34 | 291 | 6 |
|  |  |  |  |  |
| Prelingual only |  | Value | N participants | N articles |
|  | Mean score (%) | 39.49 | 14 | 2 |
|  | Mean score (SD) | 31.62 | 14 | 2 |
|  | Min range | 0.0 – 0.0 | 14 | 2 |
|  | Max range | 7.24 – 95 | 14 | 2 |
|  | Insufficient number of articles to combine further measures | | | |
|  |  |  |  |  |
| Postlingual younger adults (<76 year old), no SSD |  | Value | N participants | N articles |
|  | Mean score (%) | 74.74 | 364 | 10 |
|  | Mean score (SD) | 28.23 | 209 | 6 |
|  | Min range | 0- 59.83 | 93 | 5 |
|  | Max range | 94.0 – 100.0 | 93 | 5 |
|  | 25^th^ percentile | 58.63 | 75 | 3 |
|  | Mean improvement CI alone | 50.30 | 170 | 5 |
|  | Mean improvement best aided | 55.27 | 171 | 5 |
|  |  |  |  |  |
| Postlingual older adults ( >59 year old; range 60-93 year old), no SSD |  | Value | N participants | N articles |
|  | Mean score (%) | 71.89 | 177 | 6 |
|  | Mean score (SD) | 26.43 | 78 | 3 |
|  | Min range | 1.7 – 2.8 | 45 | 2 |
|  | Max range | 99.15 - 100 | 45 | 2 |
|  | 25^th^ percentile | 57.79 | 45 | 2 |
|  | Mean improvement CI alone | 39.29 | 100 | 3 |
|  | Mean improvement best aided | 51.86 | 86 | 2 |
|  |  |  |  |  |
| Adults with SSD |  | Value | N participants | N articles |
|  | Mean score (%) | 66.01 | 89 | 2 |
|  | Mean score (SD) | 24.42 | 16 | 1 |
|  | Min range | 0- 46.74 | 28 | 2 |
|  | Max range | 93.26 – 98.74 | 28 | 2 |
|  | 25^th^ percentile | 57.58 | 73 | 2 |
|  | Mean improvement CI alone | 23.47 | 16 | 1 |
|  | Mean improvement best aided | na | na | na |
|  |  |  |  |  |
