# Supplementary material for: Cochlear implantation outcomes in adults: A scoping review
Source: PLoS One. 2020 May 5;15(5):e0232421. doi: 10.1371/journal.pone.0232421 (PMC7199932; doi:10.1371/journal.pone.0232421)
Supplement: S5 Table — Sentence perception in noise scores across different subgroups. (DOCX) [file pone.0232421.s007.docx]

**S7 Table. Sentence perception in noise scores, CI alone.**

Postoperative sentence perception in noise scores with the implanted ear alone, across different subgroups.

| All populations  Insufficient number of articles for subgroup analyses |  | Value | N participants | N articles |
| --- | --- | --- | --- | --- |
|  |  |  |  |  |
|  | Mean score (%) | 49.78 | 454 | 12 |
|  | Mean score (SD) | 27.27 | 336 | 10 |
|  | Min range | 1. 34.0 | 158 | 8 |
|  | Max range | 20.0 -100.0 | 158 | 8 |
|  | 25^th^ percentile | 4.05 | 123 | 2 |
|  |  |  |  |  |
