# Supplementary material for: Cochlear implantation outcomes in adults: A scoping review
Source: PLoS One. 2020 May 5;15(5):e0232421. doi: 10.1371/journal.pone.0232421 (PMC7199932; doi:10.1371/journal.pone.0232421)
Supplement: S6 Table — Postoperative improvement in self-report outcome measures and change in relation to preoperative performance. (DOCX) [file pone.0232421.s008.docx]

**S8 Table. Self-reported results.**

Postoperative improvement in self-report outcome measures and change in relation to preoperative performance

|  | **Postoperative score** | | | | | | | | | **Change from pre- to postoperative** | | |
| --- | --- | --- | --- | --- | --- | --- | --- | --- | --- | --- | --- | --- |
| **Outcome measure** | **Weighted mean** | **N participants** | **N articles** | **SD** | **N participants** | **N articles** | **Median** | **N participants** | **N articles** | **Weighted mean change (range across studies)** | **N participants** | **N articles** |
| APHAB (%) [lower is better] | 39.5 | 72 | 3 |  |  |  |  |  |  | -20.12 (-8 to -25.3) | 92 | 4 |
| CAP (0-7) [higher is better] | 5.06 | 135 | 3 |  |  |  |  |  |  |  |  |  |
| GBI (-100 to + 100) [higher is better] | 50.84 | 162 | 5 | 19.17 | 87 | 3 |  |  |  |  |  |  |
| HHI (%) [lower is better] | 40.21 | 188 | 2 |  |  |  |  |  |  | -33.66 (-30.1 - -36.9) | 188 | 2 |
| HUI3 - multi (0-1) [lower is better] | 0.28 | 75 | 3 |  |  |  | 0.68 | 56 | 2 | 0.06 (0.03 - 0.24) | 86 | 4 |
| NCIQ - total (0-100) [higher is better] | 58.06 | 391 | 9 | 16.16 | 320 | 7 |  |  |  | 16.89 (0 - 28.6) | 391 | 9 |
| Oldenburg inventory (1-5) [higher is better] | 3 | 62 | 2 | 0.69 | 62 | 2 |  |  |  | 0.82 (0.75 - 0.85) | 62 | 2 |
| SSQ - total (0-10) [higher is better] | 4.97 | 124 | 5 | 1.45 | 41 | 2 | 5.94 | 24 | 2 | 2.53 (0.55 - 3.23) | 152 | 7 |
